# *Clostridium thermocellum* transcriptomic profiles after exposure to furfural or heat stress

**DOI:** 10.1186/1754-6834-6-131

**Published:** 2013-09-12

**Authors:** Charlotte M Wilson, Shihui Yang, Miguel Rodriguez, Qin Ma, Courtney M Johnson, Lezlee Dice, Ying Xu, Steven D Brown

**Affiliations:** 1 Biosciences Division, Oak Ridge National Laboratory, Oak Ridge, 37831 TN, USA; 2 BioEnergy Science Center, Oak Ridge National Laboratory, Oak Ridge, TN 37831, USA; 3 Present address: National Bioenergy Center, National Renewable Energy Laboratory, Golden, CO 80401, USA; 4 Department of Biochemistry and Molecular Biology and Institute of Bioinformatics, Computational Systems Biology Laboratory, University of Georgia, Athens, GA 30602, USA; 5 College of Computer Science and Technology, Jilin University, Changchun, Jilin, China

**Keywords:** Biomass, Recalcitrance, Inhibitor, Stress, DNA microarray, Regulation, Regulatory motif

## Abstract

**Background:**

The thermophilic anaerobe *Clostridium thermocellum* is a candidate consolidated bioprocessing (CBP) biocatalyst for cellulosic ethanol production. It is capable of both cellulose solubilization and its fermentation to produce lignocellulosic ethanol. Intolerance to stresses routinely encountered during industrial fermentations may hinder the commercial development of this organism. A previous *C. thermocellum* ethanol stress study showed that the largest transcriptomic response was in genes and proteins related to nitrogen uptake and metabolism.

**Results:**

In this study, *C. thermocellum* was grown to mid-exponential phase and treated with furfural or heat to a final concentration of 3 g.L^-1^ or 68°C respectively to investigate general and specific physiological and regulatory stress responses. Samples were taken at 10, 30, 60 and 120 min post-shock, and from untreated control fermentations, for transcriptomic analyses and fermentation product determinations and compared to a published dataset from an ethanol stress study. Urea uptake genes were induced following furfural stress, but not to the same extent as ethanol stress and transcription from these genes was largely unaffected by heat stress. The largest transcriptomic response to furfural stress was genes for sulfate transporter subunits and enzymes in the sulfate assimilatory pathway, although these genes were also affected late in the heat and ethanol stress responses. Lactate production was higher in furfural treated culture, although the lactate dehydrogenase gene was not differentially expressed under this condition. Other redox related genes such as a copy of the *rex* gene, a bifunctional acetaldehyde-CoA/alcohol dehydrogenase and adjacent genes did show lower expression after furfural stress compared to the control, heat and ethanol fermentation profiles. Heat stress induced expression from chaperone related genes and overlap was observed with the responses to the other stresses. This study suggests the involvement of *C. thermocellum* genes with functions in oxidative stress protection, electron transfer, detoxification, sulfur and nitrogen acquisition, and DNA repair mechanisms in its stress responses and the use of different regulatory networks to coordinate and control adaptation.

**Conclusions:**

This study has identified *C. thermocellum* gene regulatory motifs and aspects of physiology and gene regulation for further study. The nexus between future systems biology studies and recently developed genetic tools for *C. thermocellum* offers the potential for more rapid strain development and for broader insights into this organism’s physiology and regulation.

## Background

Processing biomass for biochemical conversion of plant cell wall polysaccharides for fuel production by fermentation requires a pretreatment step that often involves an acid hydrolysis at high temperatures [1,2]. The disadvantage of this treatment is the release of fermentation inhibitors such as the sugar degradation product furfural [3,4]. Depending on the fermentative microorganism, the release of these inhibitors can affect an organism’s ability to produce ethanol and even to grow [3,4]. A further consideration in choosing an organism for biofuel production is tolerance to fermentation end products such as ethanol. Metabolic engineering to facilitate redirection of carbon flow towards increased ethanol titer does mean the organism used for this process needs to have a high tolerance to the desired solvent.

The thermophilic anaerobe *Clostridium thermocellum* is of interest due to its ability to convert biomass cellulose to ethanol [5,6]. *C. thermocellum* produces a large extracellular protein complex called the cellulosome which is highly active on plant cell wall polysaccharides [7-9]. The native production of enzymes required for cellulose deconstruction is economically advantageous for industrial purposes, thus circumventing the costly addition of non-native hydrolytic enzymes. Recent advances for *C. thermocellum* include genome sequences for a number of strains [10-13], strategies and strains to delete genes [14-17], characterization of CRISPR elements [18], genetic and mechanistic insights into ethanol tolerance [10], as well as carbon recovery [19]. A number of systems biology studies have been conducted with *C. thermocellum* in the last several years, including; transcriptomic profiles for batch crystalline cellulose fermentations [20], chemostat growth with either cellobiose or crystalline cellulose at different dilution rates [21], proteomic analysis of cellulosomes [22,23], proteomic analysis of core metabolism [24], and an “omics” analysis of ethanol stress [25]. In addition, a genome-scale metabolic model has been developed [26] and several regulatory systems have been characterized [27-29].

In this study, we have compared the effects on the *C. thermocellum* transcriptome upon heat, or furfural exposure and compared this to a recently published dataset of ethanol exposure. This was undertaken to understand the global effects of these stressors and to contrast a physical stress such as the well characterized microbial response to high temperature with the chemical stress imposed by ethanol or furfural treatment. Ultimately by revealing how this organism is affected by these stressors, we aim to add valuable knowledge to the way this organism could be developed and utilized for applied industrial goals.

## Results

The time point in mid-exponential growth when the test fermentations were exposed to stress was designated as “time zero”. Samples were either referred to as “control” for untreated control fermentations or heat or furfural shock (or treatment) for those derived from fermentations that had an increased temperature 68°C or 3 g.L^-1^ furfural treatment respectively at “time zero”.

### *C. thermocellum* growth response

**Figure 1:**
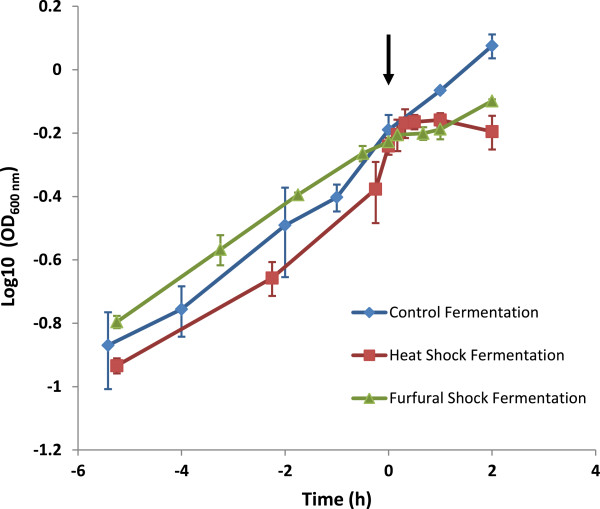
**Growth curves of untreated (control), furfural (3 g.L**^**-1**^**) or heat (68°C) treated *C. thermocellum *fermentations.** Time 0, also indicated by an arrow, is when the stress treatment was applied to the fermentation with the exponential phase preceding this event shown as negative values on the x-axis. Error bars are the standard deviation of two replicate fermentations.

The application of each shock treatment negatively influenced *C. thermocellum* growth (Figure 1). The culture turbidity of OD_600nm_ units for the experimental fermentors prior to the administration of shock treatment was similar to the control fermentors and ranged from 0.57-0.62 (Figure 1). Growth rates (1/time (h^-1^)) leading up to the administration of each stressor treatment were 0.25, 0.30, 0.26 and 0.29 for the furfural shock, heat shock, ethanol and control fermentor respectively. OD_600nm_ measurements taken periodically (up to 150 min) post shock demonstrated the negative effect each shock had on growth with averaged growth rates (1/time (h^-1^)) across the monitored 120 min stress period declining to 0.15, 0.05 and 0.07 for furfural, heat and ethanol treatment respectively relative to the relatively stable growth rate of 0.31 for the control fermentations across the same period. Growth rates for the ethanol shock experiment were calculated from data reported previously [25]. Heat shock caused a decrease in culture turbidity, although this occurred 30 min after the temperature of the fermentor reached 68°C. Furfural and ethanol both caused a plateau in growth immediately following addition of each stress, and growth began to resume in both conditions towards the end of the monitored period. Analysis of fermentation products illustrates that less than 50% of carbon consumed as cellobiose across the 120 min sampling period in the control fermentations could be recovered as acetate, ethanol or lactate (or assumed CO_2_) (Table 1). Previous studies have shown that *C. thermocellum* carbon recoveries can be variable and carbon balances can be made of products that were not measured as part of this study [19,24]. A larger proportion of carbon would likely have been recovered if the fermentations were allowed to transition to stationary phase with end point sampling once the substrate was exhausted. The batch fermentors used in this study were open systems so any carbon lost as CO_2_, or volatile products were not measured although we assume in our calculations that a mole of CO_2_ was produced per mole of acetic acid and ethanol (Table 1). Carbon recovery for furfural increased to almost 100% across the 120 min post stress period for furfural, due to a reduction in cellobiose consumption as cell growth plateaued and was coupled with a dramatic rise in lactate production relative to the control (0.3 g.L^-1^ produced in the furfural stress fermentor compared to 0.008 g.L^-1^).

**Table 1:** ***C. thermocellum *cellobiose consumption and fermentation products across each 120 min treatment period**

**Growth condition**	Cellobiose consumed* g.L^**-1**^	Acetate produced* g.L^**-1**^	Ethanol produced* g.L^**-1**^	Lactate produced* g.L^**-1**^	**% Carbon recovery†**
**Across the stress period (0–120 min post stress application)**					
Heat Stress Fermentation	1.26 (±0.39)	0.18 (±0.04)	0.06 (±0.18)	0.014 (±0.006)	30.7
Furfural Stress Fermentation	0.48 (±0.12)	0.18 (±0.01)	−0.04 (±0.004)	0.30 (±0.010)	97.7
Control Fermentation	1.35 (±0.62)	0.29 (±0.13)	0.12 (±0.04)	0.008 (±0.005)	47.0
**Across the whole experiment (post inoculation to 120 min post stress application)**					
Control Fermentation	3.02 (±0.15)	0.47 (±0.12)	0.20 (±0.01)	0.02 (±0.003)	35.02

*Values are the mean of duplicate fermentations and the standard error of the mean given in parentheses.

†Percentage carbon recovery was calculated using the following equation $R_{C2+C3}^{C}=\frac{3\times \left(\left[A\right]+\left[E\right]+\left[L\right]\right)}{6\times \left(\left[S_{o}-S_{f}\right]\right)}$ as described in [19,49] where $R_{C2+C3}^{C}$ is the carbon recovered from the C2 and C3 fermentation products. [A], [E], and [L] are the molar concentrations of acetate [A], ethanol [E], and lactate [L]. The factor of 3 in the numerator accounts for the expected formation of 1 mole of CO_2_ and/or formate produced per mole of ethanol and acetate as previously described [19]. [*S*_*o*_] is the initial substrate concentration and [*S*_*f*_] is the final substrate concentration.

No ethanol was produced during the stress period after furfural addition and acetate production dropped relative to the control. After heat stress, the cellobiose consumption did not decrease substantially relative to the control, however, the production of acetate and ethanol decreased relative to the control with some additional lactate produced, thus suggesting an alternative outlet for carbon occurred in *C. thermocellum* after this particular stress. Furfural was applied at mid-exponential phase to a final concentration of 3 g.L^-1^ and detected after its application. At the first sampling time point, 10 min post addition, 2.87 g.L^-1^ of furfural was measured (S.D. 0.04 g.L^-1^) and at the final timepoint 2.24 g.L^-1^ (S.D. 0.06 g.L^-1^) of furfural was detected. This decrease could potentially be attributed to metabolic conversion of furfural to furfuryl alcohol as demonstrated in *Cupriavidus basilensis*[30] and *Escherichia coli*[31], and evaporation may be another factor that was not accounted for in this study.

### Transcriptomic profile of *C. thermocellum* in response to stress exposure

Gene expression profiles for the control, furfural or heat-treated fermentations were generated from samples harvested at time 10, 30, 60 and 120 min post-shock using NimbleGen microarrays including an existing ethanol stress dataset [25]. Comparison to gene expression profiles derived from untreated control fermentations permitted the identification of stress induced transcriptional changes. When filtered by a significance threshold of *p <* 0.05, 3056/3067 genes were considered significantly different in at least a single time point for a given stress condition. Using a cut off of 2-fold difference in gene expression between the treated and untreated control, 779, 731 and 683 genes were affected by ethanol, furfural or heat treatment respectively during at least one time point sampled, with 1,474 genes significantly (*p* < 0.05) differentially expressed (± 2 fold relative to the control fermentations) in at least one of the stress studies (Additional file 1: Table S1). The microarray dataset has been deposited in NCBI GEO under the accession number GSE40402.

A subset of genes that were differentially expressed 120 min post heat shock and furfural shock relative to the control profiles were selected for real-time quantitative PCR (RT-qPCR) confirmation. The ethanol shock microarray was previously validated [25]. Correlation coefficient values of *R*^*2*^ = 0.96 and 0.93 were obtained for comparisons between the microarray and RT-qPCR expression profiles for heat and furfural shock respectively (Additional file 2), indicating the microarray data were of good quality.

**Figure 2:**
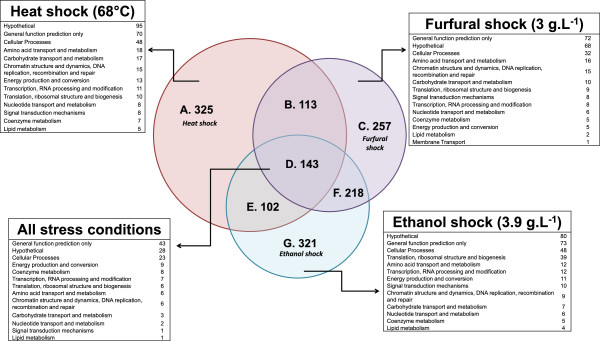
**Comparison of transcriptome response of *C. thermocellum *27405 to ethanol, furfural and heat shock relative to untreated control fermentations.** Genes were categorized into functional groups based on COG assignment and listed for those genes exclusively affected in each treatment or affected in all stresses. A through G categories for each Venn segment is used to cross reference to Additional file 1: Table S1 as listed in the column titled Venn diagram code.

A comparison of the affected genes under each of the stress conditions is summarized in Figure 2 with genes categorized by Clusters of Orthologous Groups (COG) [32]. One hundred and forty three genes were differentially expressed under all conditions tested. The majority of these shared genes (71), were assigned to the General Function Prediction or Hypothetical Protein Prediction categories. The largest category with a functional prediction was Cellular Processes with twenty-three genes assigned to this group. Within Cellular Processes nine genes had predicted functions in Inorganic Iron Transport and Metabolism and five were involved in Cell Envelope Biogenesis. Additional file 1: Table S2 lists the 143 genes differentially expressed in all the stress conditions. Consistent up-regulation occurred in the eleven genes between Cthe_2532 and Cthe_2542 that include genes encoding components of a putative sulfate transporter (Cthe_ 2532, 2533, 2534) and genes related to the sulfur assimilatory pathway. Conversion of sulfite to the biologically relevant sulfide may be catalyzed by the enzyme sulfite reductase (Cthe_2541) in a reaction dependent on three molecules of NADPH. Sulfite reductase requires siroheme as a cofactor [33] and other genes within this genomic region encode proteins involved in vitamin B12 metabolism, porphyrin and siroheme biosynthesis. Related to vitamin B12 metabolism, were the genes Cthe_2784-2789 encoding methyltransferases, ferredoxin and cobalamin B12 binding proteins up-regulated after all treatments, which suggests an increased need for vitamin B12 and corrinoid containing proteins upon exposure to each of these stresses. Other genes affected by the three stress treatments with possible redox balance functions included two NADH dehydrogenases (Cthe_0429, Cthe_3023) and an annotated CO dehydrogenase (Cthe_2801). The CO dehydrogenase formed a putative operon with an ABC transporter of unknown function (Cthe_2802-2804) and was affected by all stress treatments. Genes encoding the molecular chaperones DnaK (Cthe_1322) and GrpE (Cthe_1323) were up-regulated after all stressor treatments.

A subset of genes that were most differentially expressed (ten up regulated and ten down regulated genes) for each of the stress conditions is given in Table 2. Genes were ranked based on differential gene expression for each of the time points and the ten highest and ten lowest for each time point were given a score, lowest being the genes most down-regulated and highest being genes most up-regulated. The first 10 min time period for heat stress was excluded in this analysis due to few genes passing the significance threshold of *p* < 0.05. This is most likely due to a delay in the cells response to heat stress as seen from the growth curve where the fermentation exhibits an effect from 30 min post stress application. Gene expression patterns after heat shock include the up-regulation of central carbon metabolic genes such as Cthe_3116 encoding a mannose-6-phosphate isomerase and Cthe_2449 encoding one of the six *C. thermocellum* ATCC 27405 phosphoglycerate mutase enzymes. Phosphoglycerate mutase was shown to be highly expressed as part of the *E. coli* heat shock response and was suggested to reflect the increased energy demand for the synthesis of heat shock proteins [34]. Cthe_3125 encoding the heat shock protein Hsp20 was up-regulated after furfural shock and was one of the most highly up-regulated genes after heat shock, with the highest level of expression occurring 30 min after the temperature stabilized at 68°C. Other heat specific responses were a down-regulation of the gene Cthe_0665 and Cthe_0666 encoding the membrane bound HflK and HflC proteins. Homologs of the latter two proteins have a role in sensing heat stress in *E. coli* and triggering a heat shock response via the regulation of the FtsH protease [35]. A gene (Cthe_2276) encoding an FtsH protein was down-regulated in heat stress thus suggesting at least one signaling cascade for sensing environmental stress in *C. thermocellum*.

**Table 2:** Subset of genes with the highest (n = 10) and lowest (n = 10) differential expression to each of the treatments compared to control fermentations

**Locus tag**	**Functional annotation**	**Ratio: control – stress**^**a**^				**Venn code**
		**10 min**	**30 min**	**60 min**	**120 min**	
**Furfural stress**						
Cthe_0211	Glycoside hydrolase family 16	**−2.22**	**−3.56**	**−2.41**	**−2.87**	D
Cthe_1643	Phage-associated protein	**−2.12**	**−4.49**	**−2.41**	**−2.77**	F
Cthe_1644	Hypothetical protein	**−2.26**	**−4.07**	**−2.27**	**−2.79**	F
Cthe_2531	Sulfate ABC transporter, periplasmic sulfate-binding protein	**−1.87**	**−3.19**	**−3.58**	**−3.99**	F
Cthe_2532	Sulfate ABC transporter, inner membrane subunit CysT	**−1.90**	**−2.92**	**−3.07**	**−3.55**	D
Cthe_2533	Sulfate ABC transporter, inner membrane subunit CysW	**−1.77**	**−2.82**	**−3.29**	**−3.46**	D
Cthe_2535	Adenylylsulfate reductase, thioredoxin dependent	**−1.54**	**−2.86**	**−3.27**	**−3.45**	D
Cthe_2962	Oligopeptide/dipeptide ABC transporter, ATPase subunit	**−1.87**	**−3.04**	**−2.15**	**−2.83**	F
Cthe_2963	Oligopeptide/dipeptide ABC transporter, ATPase subunit	**−1.96**	**−3.33**	**−2.15**	**−2.59**	F
Cthe_3125	Heat shock protein Hsp20	**−2.60**	**−3.21**	**−2.49**	**−2.30**	B
Cthe_0422	Redox-sensing transcriptional repressor rex	**2.98**	**3.47**	**2.81**	**3.48**	B
Cthe_0424	Aminoglycoside phosphotransferase	**1.98**	**3.14**	**2.23**	**2.93**	B
Cthe_0425	Hypothetical protein	**2.49**	**4.07**	**3.23**	**3.84**	D
Cthe_0426	Fe-S cluster domain protein	**2.47**	**3.86**	**2.82**	**3.60**	B
Cthe_0427	Stage II sporulation protein E	**2.31**	**4.20**	**2.46**	**3.42**	D
Cthe_0428	NADH dehydrogenase (ubiquinone) 24 kDa subunit	**3.08**	**5.49**	**3.05**	**4.61**	F
Cthe_0430	Hydrogenase, Fe-only	**1.79**	**4.83**	**2.90**	**4.06**	F
Cthe_0431	Hypothetical protein	**2.24**	**5.64**	**3.15**	**4.74**	D
Cthe_0943	Hypothetical protein	**2.53**	**3.30**	**2.63**	**2.86**	C
Cthe_0944	SMC domain protein	**2.20**	**2.88**	**2.32**	**2.63**	C
**Heat stress**						
Cthe_1309	Radical SAM domain protein	0.14	**−1.94**	**−2.37**	**−2.14**	A
Cthe_1743	Protein of unknown function DUF955	**−1.89**	**−2.77**	**−2.58**	**−2.72**	D
Cthe_1746	Hypothetical protein	−0.33	**−1.99**	**−2.98**	**−1.82**	D
Cthe_1851	Protein of unknown function DUF1113	−0.30	**−2.84**	**−2.29**	**−1.76**	D
Cthe_2448	ABC-type transporter, integral membrane subunit	**−0.40**	**−2.82**	**−2.19**	**−1.66**	A
Cthe_2449	Phosphoglycerate mutase	−0.03	**−2.44**	**−2.04**	**−1.84**	A
Cthe_3054	Hypothetical protein	0.01	**−2.75**	**−2.03**	**−2.33**	B
Cthe_3112	Glycosidase related protein	**−0.71**	**−2.61**	**−2.52**	**−1.71**	B
Cthe_3116	Mannose-6-phosphate isomerase, class I	−0.13	**−2.78**	**−2.84**	**−2.11**	B
Cthe_3125	Heat shock protein Hsp20	−0.06	**−4.07**	**−2.80**	**−2.74**	B
Cthe_0323	Hypothetical protein	−0.44	**1.72**	**2.06**	**1.49**	B
Cthe_0539	ABC transporter related	−0.23	**2.10**	**2.18**	**2.23**	B
Cthe_0665	HflK protein	0.13	**2.47**	**2.95**	**2.03**	A
Cthe_0938	Regulatory protein DeoR	−0.20	**2.22**	**2.53**	**1.96**	D
Cthe_1390	Alpha/beta hydrolase fold	**1.26**	**3.66**	**2.91**	**2.75**	D
Cthe_1921	Transcriptional regulator PadR family protein	**1.03**	**1.77**	**2.94**	**2.65**	A
Cthe_1922	Hypothetical protein	0.56	**1.41**	**2.64**	**2.43**	A
Cthe_2266	Vacuolar H+transporting two-sector ATPase F subunit	−0.03	**1.49**	**2.58**	**1.87**	E
Cthe_2956	Hypothetical protein	0.54	**1.86**	**1.96**	**1.61**	B
Cthe_2957	Hypothetical protein	0.53	**2.01**	**1.56**	**1.57**	B

^a^Negative values indicate greater gene expression in the treatment samples while positive values indicate lower gene expression in the treatment samples compared to the control fermentations. Boldface indicates the differential gene expression was statistically significant (*p* < 0.05).

A region of genes related to redox balance (Cthe_0422-0432) were the most down-regulated genes in response to furfural exposure including the redox response regulator Rex (Cthe_0422), an NADH hydrogenase (Cthe_0427), and a Fe-hydrogenase (Cthe_0430). Within this genomic region is the alcohol dehydrogenase gene (Cthe_0423), which has been identified as important in *C. thermocellum* ethanol tolerance as a shift in cofactor specificity occurs from NADH to NADPH [10] indicating cellular redox status is associated with the functioning of this enzyme. Two of the most down-regulated genes after furfural shock were Cthe_0943 and Cthe_0944, which were part of a larger region of Cthe_0940-0953 specifically down-regulated after furfural treatment.

Ethanol stress effects on gene regulation in *C. thermocellum* 27405 have been described [25]. Briefly, genes related to nitrogen metabolism were up-regulated including the subunits of the urease enzymes and related accessory proteins in the genomic region of Cthe_1816-1823, glutamine synthetase (Cthe_1539), and glutamate synthase (Cthe_0198). While, ribosomal proteins and acetate kinase were down-regulated in response to ethanol as previously noted [25].

**Figure 3:**
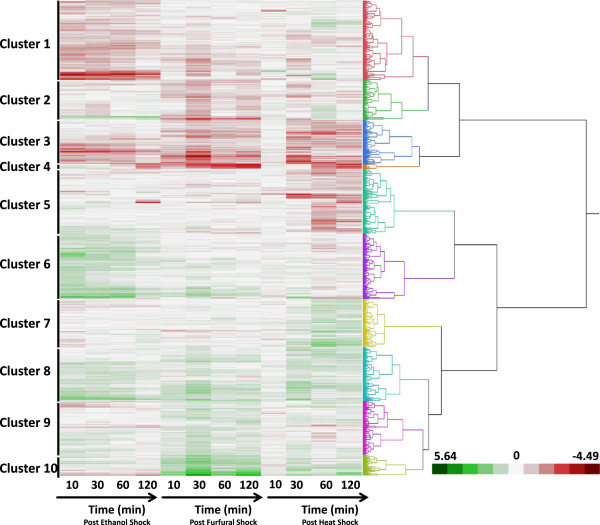
**Hierarchical clustering of the 1,474 genes significantly (*p *> 0.05) differentially expressed in the treatments relative to the control *C. thermocellum *fermentations over the four sampling time points analyzed in this analysis.** Genes were grouped into ten clusters based on responses to ethanol, furfural and heat treatments. Red indicates higher expression relative to the control, green represents down regulation of gene expression relative to the control.

A hierarchical clustering of the 1,474 genes that were significantly differentially expressed by at least 2-fold between a stress condition and the control in at least a single time point was divided into 10 clusters (Figure 3, Additional file 1: Table S1). Clusters 1 through 4 grouped those genes that were in general up-regulated after exposure to stress while Cluster 8 included the majority of genes consistently down-regulated after all the treatments. Cluster 4 consisted of sixteen genes up-regulated consistently after furfural exposure and in the latter time points of heat and ethanol exposure including those related to sulfur transport and assimilation and Cthe_2801-2804 encoding a carbon monoxide dehydrogenase subunit and transporter as mentioned above. Cluster 10 featured fifty-nine genes including the genes Cthe_0422-0432 mentioned above, that were subjected to a rapid and sustained down-regulation after exposure to furfural. Heat shock tended to result in gene down-regulation while ethanol exposure had little effect on the Cluster 10 genes (Figure 3, Additional file 1: Table S1).

### Cellulosome genes

Fifty-six genes associated with the cellulosome were differentially regulated in at least one of the three stress conditions (Additional file 1: Table S1). Seven cellulosome genes were consistently up-regulated in at least one time point after stress including the glycoside hydrolases (Cthe_0745 Cthe_2590). These were recently shown to be affected by growth rate where higher expression from these loci occurred at lower growth rates [21]. These expression patterns would therefore reflect the decreased growth rates in the batch fermentations once stress had been applied. It is plausible that other cellulosome related genes with consistent increased expression across the stress treatments were also a growth rate effect rather than a reaction to the application of each of the stressors.

### Transcriptional regulators

Sixty-one transcriptional regulators were differentially expressed in at least one stress condition (Additional file 1: Table S3). Four regulators, Cthe_1619, Cthe_1745, Cthe_2808 and Cthe_2969 were consistently up-regulated in the three stress conditions relative to the control and are located next to genes also up-regulated. Cthe_1745 was up-regulated in all conditions and located in the region Cthe_1743-1750. This region was up-regulated after all treatments relative to the control fermentations and includes genes coding DNA mismatch repair enzymes, restriction endonucleases and genes functionally annotated as hypothetical. Cthe_2808, a LacI repressor was up-regulated after all stresses and its repression in *C. thermocellum* has been shown to be alleviated by laminaribiose [27,36]. The genes (Cthe_2807 and Cthe_2809) that the LacI regulator is known to repress were up-regulated after each of the stress treatments, although these genes were just below the 2-fold threshold for differential expression after furfural treatment. This suggests that the expression of these genes can be influenced by other environmental stimuli besides the presence of laminaribiose.

Other regulators of interest included Cthe_2524 which is located upstream of the sulfate transporter mentioned above and was down-regulated after all stress treatments. The putative phosphate uptake regulator PhoU, encoded by Cthe_1601, was up-regulated under ethanol stress, down-regulated after heat stress and unaffected by furfural treatment and is located beside the phosphate transport components. The housekeeping sigma factor sigma 70 (Cthe_2521), required for transcriptional initiation, was down-regulated under all stress conditions. The gene Cthe_1792, encoding a CtsR regulator or Firmicute transcriptional repressor of Class III stress genes was up-regulated 120 min post heat treatment.

### Regulatory motif analysis

A motif identification strategy was undertaken to identify *cis* regulatory motifs in the promoter regions of genes identified as differentially expressed in at least one of the stress analyses. In total, 120 *cis* regulatory motifs were identified by a phylogenetic comparison framework using 39 *Clostridium* strains and 17,328 different *C. thermocellum* ATCC27405 genomic regions of interest were identified (Additional file 1: Table S4). The 120 motifs are further clustered into fifty-four groups regarding their patterns’ similarity (Motif cluster ID column, Additional file 1: Table S4) and were used for a co-expression analysis to identify those genes in the stress studies that potentially formed an operon and were downstream from a particular motif. Six motifs (Additional file 3: Table S5) were located in the promoter regions of genes that were co-regulated and investigated further to identify potential regulators that had responded to heat, furfural or ethanol stress. We highlight several motifs below and provide the others as supplemental material.

Genes with the mapped Motif 29 in general responded to stress treatments similarly, while genes with Motif 51 tended to respond to ethanol stress more than heat or furfural. Motif 29 (Additional file 3: Table S5) appeared upstream of 14 genes, five of which encoded a putative sulfate transporter (Cthe_2531-2535) that appeared to be co-expressed and potentially form an operon. Motif 29 was most similar to a sequence recognized by IscR (Iron sulfur cluster Regulator) from Rickettsiales. A BLASTP search using the IscR amino acid sequence from *Clostridium acetobutylicum* ATCC 824 as the query sequence against *C. thermocellum* 27405 found one of the top hits (ID 70/208, 99% sequence coverage) in *C. thermocellum* as Cthe_2524, currently annotated as a BadM/Rrf2 family transcriptional regulator and located upstream of the sulfate transporter Cthe_2531-2535. Cthe_2524 was down-regulated after all stress treatments and may have a negative regulatory role in the transcription of the downstream sulfate transport genes. Interestingly, Motif 29 occurs upstream of a gene encoding Hsp20 (Cthe_3125) and could indicate the sulfate transport system up-regulation is part of a general stress response coordinated with the classical heat shock response. Motif 51 (Additional file 3: Table S5) was identified upstream of 16 genes, encompassing three regions of putative co-expression. Two cellulosome related dockerins (Cthe_0729 and Cthe_0246) and a glycosyl transferase were both induced by ethanol treatment and had Motif 51 upstream of the transcriptional start site. The consensus regulatory motif sequence is most likely recognized by a member of the AraC family of regulators with one AraC regulator (Cthe_2634) down-regulated within 10 min after ethanol stress.

## Discussion

This study sought to investigate the effect of furfural and heat stress on the physiology of the candidate CBP organism *C. thermocellum* and compare this to a previously published dataset based on ethanol stress [25]. 

Under the conditions used in this study, both heat and furfural stress caused a reduction in the fermentation growth rate compared to the untreated control and a decrease in the main fermentation products ethanol and acetate occurred as a result (Table 1). Acetyl-CoA is a branch point in *C. thermocellum* metabolism where carbon flow is directed towards either of two main fermentation products, acetate or ethanol [6]. The decreased growth of the cells following stress treatment likely reflects the impact to cellular physiology via a reduction in available cellular ATP as the reactions catalyzed by phosphotransacetylase and acetate kinase generates ATP and acetate [6]. Phosphotransacetylase (PTA; Cthe_1029) and acetate kinase (Cthe_1028) were down-regulated after all treatments, although PTA was not significantly down-regulated after heat shock (Additional file 1: Table S1). Acetyl-CoA can be converted to acetylaldehyde by the action of aldehyde dehydrogenase encoded by Cthe_2238 [20,24] or the activity of a bifunctional alcohol/aldehyde dehydrogenase (Cthe_0423) [10]. The former gene was not differentially regulated after heat shock or furfural shock; however Cthe_0423, encoding the enzyme capable of catalyzing both acetyl-CoA to acetylaldehyde and acetylaldehyde to ethanol was dramatically down-regulated after furfural shock from 10–120 min post shock by log_2_ ratios ranging from 1.91 to 2.60 (in Additional file 1: Table S1). By 120 min post heat shock, Cthe_0423 was down-regulated by a log_2_ ratio of 2.57 (Additional file 1: Table S1). These changes of expression are reflected in the amount of ethanol detected across the stress period (Table 1). No net ethanol was produced after furfural treatment and an apparent reduction occurred after heat stress. The detectable levels for ethanol after heat treatment did vary and we cannot rule out the effect of increased evaporation on ethanol concentrations as the temperature of the fermentor was raised. Lactate, typically a minor fermentation product was dramatically increased after furfural treatment (Table 1) despite no differences identified in the expression of the gene encoding lactate dehydrogenase (Cthe_1053) under this condition (Additional file 1: Table S1). The pathway to lactate has no net loss of carbon to CO_2_, hence the increased proportion of carbon recovery compared to the control fermentations, and it also generates an NAD which may help rebalance electron flow after furfural stress. The application of fermentation end-products can induce shifts in *C. thermocellum* strain ATCC 27405 metabolism and corresponding enzyme levels do not necessarily compare with differences in end-product yields [37], as was also the case for some key genes in this study.

Patterns of differential expression in the shock relative to the control indicated that the response to furfural is rapid and is maintained for a prolonged period of time. Previous work in *Saccharomyces cerevisiae* has shown that furfural tolerance requires a functional pentose phosphate pathway, and causes an accumulation of reactive oxygen species [38,39]. The redox status of the cell after furfural exposure is likely to be considerably disturbed and some of the most down-regulated genes after exposure to furfural were those in the genomic region around the gene (Cthe_0423) encoding the NADH dependent AdhE [10] and the Rex transcriptional repressor (Cthe_0422). Growth inhibition caused by furfural exposure has been shown to result from NAD(P)H depletion as the reduction of furfural to the less toxic furfuryl alcohol is catalyzed by NAD(P)H dependent oxidoreductases on NAD(P)H, thus competing with NAD(P)H dependent alcohol dehydrogenases and impeding alcohol production [40,41]. This is a likely explanation for the increased lactate production and the complete halt in ethanol production when *C. thermocellum* was treated with furfural. Furfural toxicity effects have also been demonstrated to impact in the assimilation of sulfur, as sulfite reductase is reliant on NADPH, which leads on to effects in the biosynthesis of cysteine and methionine [40]. The *C. thermocellum* Cthe_2801 gene encodes a putative carbon monoxide dehydrogenase and this enzyme may play a role in dealing with redox imbalance associated with the oxidative stress from furfural exposure, or the more extreme stress imposed by the conditions used in this study. One report has shown that CO inhibits *C. thermocellum* hydrogenases and can increase the ratio of ethanol to acetate production [42]. However, no evidence exists as to whether *C. thermocellum* can utilize the substrates of this enzyme, CO or the reverse reaction, CO_2_ as a carbon source.

The effect of raising the temperature of the fermentor from 58°C to 68°C resulted in a drop in culture turbidity 30 min after the temperature was stabilized at the higher temperature (Figure 1). Heat shock proteins are conserved and in *B. subtilis* the heat shock response genes are split into six classes, four of which I, III, IV and V were affected by the stress treatments (See review [43]). An induction of the *C. thermocellum* genes homologous to the *B. subtilis* Class I heat shock response genes occurred after all stress treatments. These included the genes encoding DnaK (Cthe_1322), the GroES domain protein (Cthe_2445), and genes in the *dnaK* operon (Cthe_1321-Cthe_1324). The *dnaK* operon in *C. thermocellum* has a similar architecture to that in *B. subtilis*[43] and includes the genes encoding HrcA repressor, GrpE, DnaK, DnaJ. Class III heat stress response genes are regulated by the CtsR regulator. The CtsR regulator in *C. thermocellum* 27405 is most likely encoded by the gene Cthe_1792, currently annotated as the Firmicute transcriptional repressor of class III stress genes (Additional file 1: Table S1). This gene was up regulated 120 min after heat stress (Additional file 1: Table S1). Interestingly, knockout of the *ctsR* gene in *Lactobacillus sakei* bypassed the lag phase during fermentation [44] and could provide a direction for metabolic engineering of the *C. thermocellum* strain. The Class IV heat shock regulon has a single member, HtpG/Hsp90. The gene Cthe_0550 is annotated as encoding Hsp90 and was up regulated after all stress treatment (Additional file 1: Table S1). Overexpression of *htpG* was recently shown to enhance butanol tolerance and eventual strain adaptation to butanol in *C. acetobutylicum*[45] and could provide a route for enhanced solvent resistance in *C. thermocellum.*

## Conclusions

In summary, transcriptome analyses revealed a global view of the responses of the *C. thermocellum* to the challenge of two stressors, furfural and heat, for the first time. Many genes were considered differentially expressed in response to two or more of the stress exposures, and 325, 257, and 321 genes were considered responsive to heat, furfural, or ethanol stress specifically using the stringency criteria applied in this study. This study suggests the involvement of *C. thermocellum* genes with functions in oxidative stress protection, electron transfer, detoxification, sulfur and nitrogen acquisition, and DNA repair mechanisms in the stress responses and the use of different regulatory networks to coordinate and control adaptation. This study has identified *C. thermocellum* gene regulatory motifs and aspects of physiology and gene regulation for further study.

## Methods

### Controlled batch fermentations

*C. thermocellum* ATCC27405 was cultured in MTC medium with 5 g.L^-1^ final concentration of cellobiose as the carbon source at 58°C as described previously [46,47]. For fermentor inoculation *C. thermocellum* 27405 was grown overnight in 50 mL MTC, which was used to inoculate a seed fermentor as described previously [25]. The seed fermentor was used to inoculate the experimental batch fermentors to a starting OD_600nm_ of approximately 0.1. Batch fermentations were performed in 7.5-L BioFlo110 bioreactors (New Brunswick Scientific, NJ) fitted with agitation, pH and temperature probes and controls with a total volume of approximately 4.0 L of MTC medium [48]. Fermentors were sparged with filter-sterilized N_2_ gas to maintain anaerobic conditions. During fermentations, the agitation rate of the vessel was maintained at 300 rpm and pH was maintained at pH 7.0 by automatic titration with 3 N NaOH as described previously [25].

Fermentations were conducted for the wild-type *C. thermocellum* controls (no added stress), a 3.9 g.L^-1^ ethanol shock treatment, a 3 g.L^-1^ furfural shock or a 68°C heat shock in duplicate. The heat shock increase in temperature from 58°C occurred over a five minute period and the first time point sample was taken once the culture temperature had reached 68°C. Growth was monitored by measuring OD_600nm_ with a model 8453 spectrophotometer (Hewlett-Packard, CA). Samples were harvested at approximately mid-exponential phase (OD_600nm_ ~ 0.5) and at different time points post treatment.

### Fermentation product analyses with high-performance liquid chromatography (HPLC)

HPLC analysis was used to measure the extracellular metabolite concentration of cellobiose, acetate, lactate, ethanol and furfural in 0.2 μm-filtered samples taken at different time points during fermentation, as described previously [48]. The fermentation samples were acidified with 2 M sulfuric acid, separated and quantified by HPLC using a LaChrom Elite System (Hitachi High Technologies America, Inc., CA). Analysis was performed with the oven (Model L-2350) set at 60°C, and a pump (Model L-2130) set with a flow rate of 0.5 mL.min^-1^ in 5 mM H_2_SO_4_ as described previously [48]. The run time for each sample was set for 35 min or extended to 80 minutes for 20-fold diluted samples for measurement of furfural (Injector Model L-2200). Eluted compounds were registered and quantified by a refractive index detector (Model L-2490) interfaced to a computer. Soluble fermentation products were identified by comparison with retention times and peak areas of corresponding standards. Metabolites were separated on an Aminex HPX-87H, 300 × 7.8 mm column (Bio-Rad, CA). Percentage carbon recoveries were calculated based on cellobiose consumed and the amount of ethanol, acetate and lactate produced [49].

### RNA extraction and ds-cDNA synthesis

Cells were harvested in two 50 ml aliquot samples taken from controlled batch fermentations by centrifugation, snap frozen and stored at −80°C. Cell pellets from one tube were resuspended in TRIzol reagent (Invitrogen, CA), disrupted and RNA extracted as described previously [25]. Cell lysis involved bead beating with 0.25 g of lysis beads from an UltraClean Microbial RNA Kit (MO BIO Laboratories, Inc, CA) at 6,500 rpm for three 20 s treatments in a Precellys 24 high-throughput tissue homogenizer (Bertin Technologies, Montigny-le-Bretonneux, France). Cell lysates were transferred to fresh tubes and purified as described previously [25,48]. Total RNA preparations were DNaseI treated (Ambion, TX) and purified using a RNeasy mini kit (Qiagen, CA). RNA quantity was determined by NanoDrop ND-1000 spectrophotometer (NanoDrop Technologies, DE) and RNA quality was assessed with Agilent Bioanalyzer (Agilent, CA). Purified RNA of high quality was used as the template to generate ds-cDNA using Invitrogen ds-cDNA synthesis kit according to the manufacturers protocols (Invitrogen, CA).

### Microarray sample labeling, hybridization, scan, and statistical analysis of array data

The ds-cDNA was labeled, hybridized and washed according to the NimbleGen protocols as described previously [25]. Hybridizations were conducted using a 12-bay hybridization station (BioMicro Systems, Inc., UT) and the arrays dried using a Maui wash system (BioMicro Systems, Inc.). Microarrays were scanned with a Surescan high-resolution DNA microarray scanner (5 μm) (Agilent Technologies, CA), and the images were quantified using NimbleScan software (Roche NimbleGen, IN). Raw data was log_2_ transformed and imported into the statistical analysis software JMP Genomics 6.0 software (SAS Institute, NC). The data of the three stress studies and the controls were normalized together using a single round of the LOESS normalization algorithm within JMP Genomics and distribution analyses were conducted before and after normalization as a quality control step. An ANOVA was performed in JMP Genomics to determine differential expression levels between conditions and time points using the False Discovery Rate (FDR) testing method (*p* < 0.05). One way Hierarchical clustering of the ratios (Control vs. Stress) of significantly differentially expressed genes were performed in JMP Genomics using the default settings of ten clusters.

### Real-Time quantitative-PCR (RT-qPCR) analysis

Microarray data were validated using real-time qPCR, as described previously [25]. Genes representing a range of gene expression values based on microarray hybridizations were analyzed using qPCR from cDNA derived from different time point samples. Oligonucleotide sequences of the primers targeting the genes selected for qPCR analysis are listed in Additional file 4: Table S4.

### Regulatory motif analysis

Genes that were considered significantly differentially expressed by microarray were analyzed by BoBro and a motif analysis toolkit to identify potential *cis* regulatory motifs [50,51]. These putative motifs were evaluated by a phylogenetic footprinting framework utilizing 39 *Clostridium* genome sequences available in public databases. This identified 120 putative motifs that were well conserved across the strains and were thus more likely to be actual *cis* regulatory elements. Furthermore, using the clustering algorithm MCL [52], the 120 motifs were clustered into 54 groups based on the similarity score between any pair of motifs (use 0.45 as the similarity score cutoff). For each one out of the 54 motif patterns, the co-expression property of downstream operons was assessed by a popular biclustering algorithm, QUBIC [48]. The motif consensus sequences were mapped to known prokaryotic *cis* regulatory elements databases [53] to determine whether the predicted motif patterns were real *cis* regulatory elements in other prokaryotic genomes.

## Competing interests

The authors declare that they have no competing interests.

## Authors’ contributions

SY, CW and SDB conceived and designed the study; SY, CW and MR conducted the fermentations; SY, CW, and SDB analyzed the microarray data, CMJ and CW generated microarray and RT-qPCR data; QM, YX and CW generated and conducted regulatory motif analyses; SY, CW and MR carried out the metabolite analysis; LD carried out furfural growth studies; SY, CW, QM, YX, and SDB analyzed data and wrote the manuscript. All authors read and approved the final manuscript.

## Supplementary Material

Additional file 1: Table S1. Genes significantly (*p* < 0.05) differentially expressed in the treatments relative to the control *C. thermocellum* fermentations over the four sampling time points analyzed in this analysis. **Table S2.** Genes that were differentially expressed in all stress conditions. **Table S3.** Genes encoding transcriptional regulators that were significantly (*p* < 0.05) differentially expressed in the treatments relative to the control *C. thermocellum* fermentations over the four sampling time points analyzed in this analysis. **Table S4.** Details of the locations in the genome of *C. thermocellum* the 120 cis motifs, identified by phylogenetic comparison of *Clostridium* spp. Target sequences clustered into fifty four groups 0–53 identified in the Motif cluster ID column. Included are columns detailing the effect of each stress treatment on the genes predicted to be under the control of the promoters of interest, 1 = differential gene expression, 0 = no differential gene expression. Motif clusters highlighted in bold are those six motifs that were statistically significant regarding co-regulated genes. Click here for file

Additional file 2: **Microarray validation by RT-qPCR.** Comparison of gene expression profiles by microarray and RT-qPCR 120 min after *C. thermocellum* was treated with 3 g.L^-1^ furfural or exposed to 68°C. Gene expression ratios from the microarray and RT-qPCR were log_2_ transformed and plotted against each other. The primer sequences are listed in Additional file 4: Table S6. Click here for file

Additional file 3: Table S5. Six motifs were located in the promoter regions of genes that were co-regulated and investigated further to identify potential regulators that had responded to heat, furfural or ethanol stress. Columns in this table are as follows: Motif Cluster ID corresponds to Additional file 1: Table 4 column of the same name; Sequence of logo of predicted DNA Binding site is the consensus sequence of this motif in *C. thermocellum*; Regulator name is the best match in available databases that recognizes that motif sequence; Optimal offset, The offset of the query motif to the matched motif in the optimal alignment; *p*-value, The probability that the match occurred by random chance according to the null model; E value, The expected number of false positives in the matches up to this point; q value The minimum False Discovery Rate required to include the match; Overlap, The number of letters that overlapped in the optimal alignment; Query consensus, the *C. thermocellum* consensus sequence (as per logo) for particular motif; Target consensus, sequence identified by proposed regulator in Regulator Name column. Click here for file

Additional file 4: Table S6. Primer sequences used for Real Time qPCR validation of the microarray results. Click here for file
