# Supplementary figures and images for: Clostridium thermocellum transcriptomic profiles after exposure to furfural or heat stress
Source: Biotechnol Biofuels. 2013 Sep 12;6:131. doi: 10.1186/1754-6834-6-131 (PMC3848806; doi:10.1186/1754-6834-6-131)

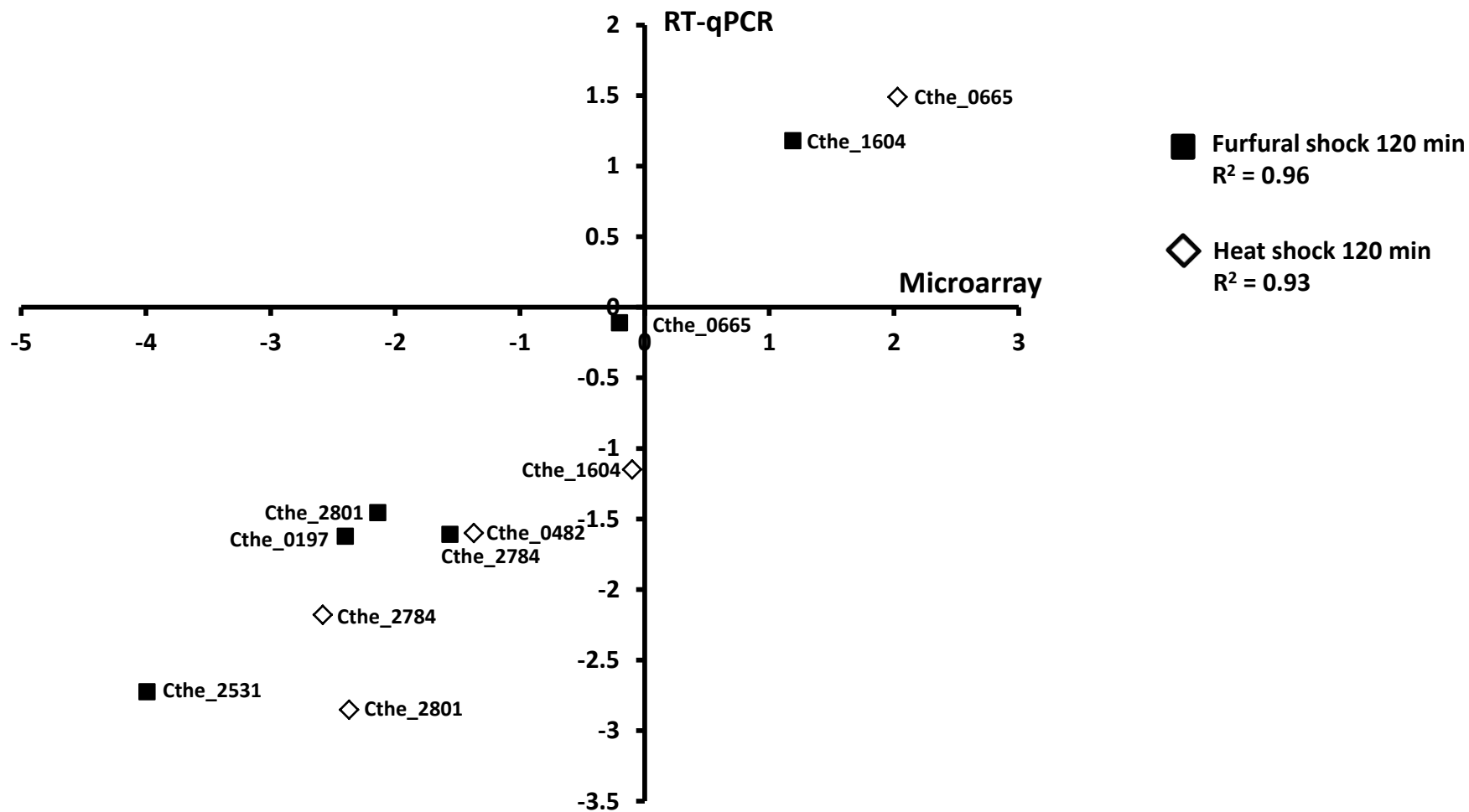

Supplement: Additional file 2: — Microarray validation by RT-qPCR. Comparison of gene expression profiles by microarray and RT-qPCR 120 min after C. thermocellum was treated with 3 g.L-1 furfural or exposed to 68°C. Gene expression ratios from the microarray and RT-qPCR were log2 transformed and plotted against each other. The primer sequences are listed in Additional file 4: Table S6. [file 1754-6834-6-131-S2.pdf]
