# Supplementary material for: Clostridium thermocellum transcriptomic profiles after exposure to furfural or heat stress
Source: Biotechnol Biofuels. 2013 Sep 12;6:131. doi: 10.1186/1754-6834-6-131 (PMC3848806; doi:10.1186/1754-6834-6-131)
Supplement: Additional file 3: Table S5. — Six motifs were located in the promoter regions of genes that were co-regulated and investigated further to identify potential regulators that had responded to heat, furfural or ethanol stress. Columns in this table are as follows: Motif Cluster ID corresponds to Additional file 1: Table 4 column of the same name; Sequence of logo of predicted DNA Binding site is the consensus sequence of this motif in C. thermocellum; Regulator name is the best match in available databases that recognizes that motif sequence; Optimal offset, The offset of the query motif to the matched motif in the optimal alignment; p-value, The probability that the match occurred by random chance according to the null model; E value, The expected number of false positives in the matches up to this point; q value The minimum False Discovery Rate required to include the match; Overlap, The number of letters that overlapped in the optimal alignment; Query consensus, the C. thermocellum consensus sequence (as per logo) for particular motif; Target consensus, sequence identified by proposed regulator in Regulator Name column. [file 1754-6834-6-131-S3.pptx]

## Slide 1
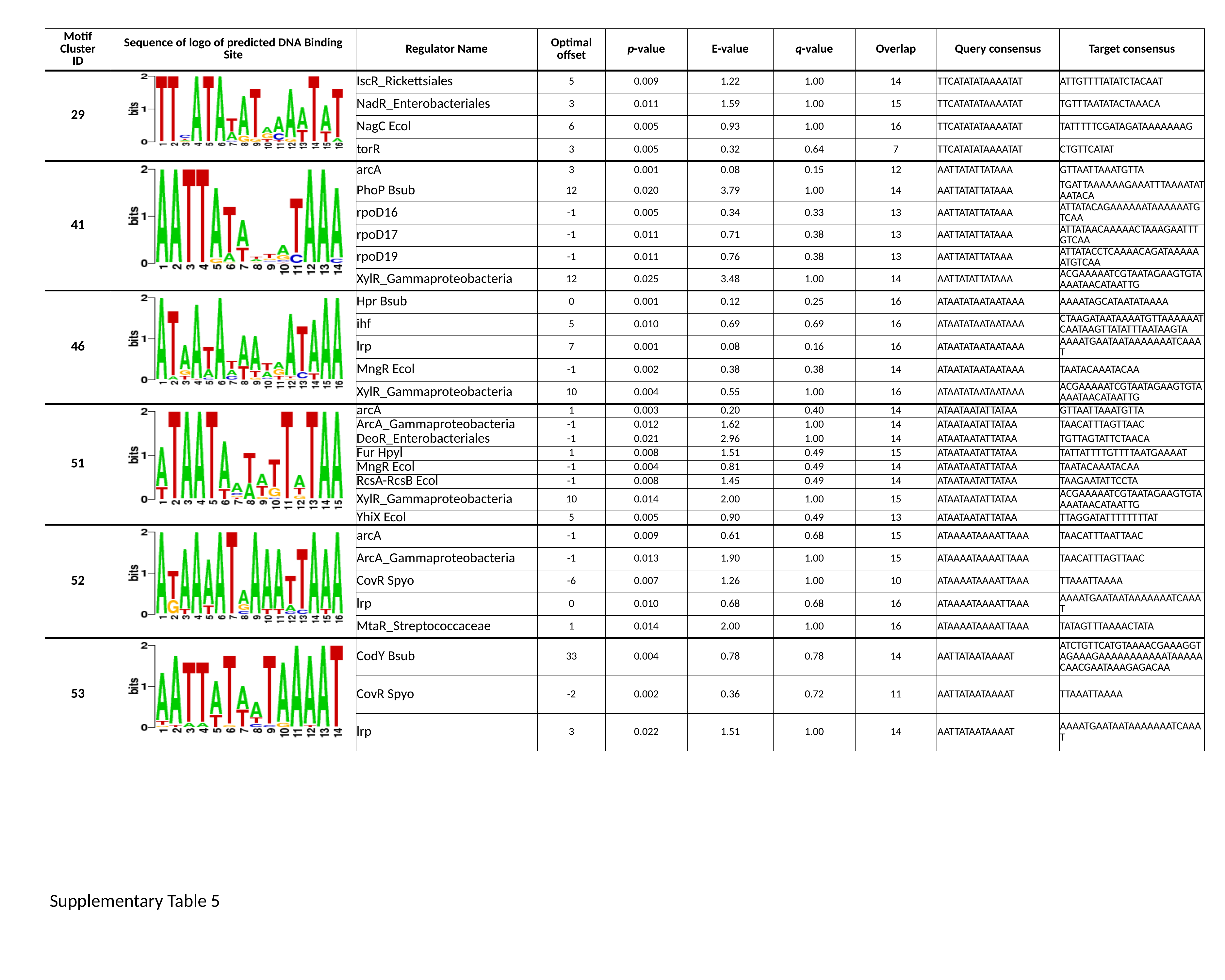

| Motif Cluster ID | Sequence of logo of predicted DNA Binding Site | Regulator Name | Optimal offset | p-value | E-value | q-value | Overlap | Query consensus | Target consensus |
| --- | --- | --- | --- | --- | --- | --- | --- | --- | --- |
| 29 | | IscR\_Rickettsiales | 5 | 0.009 | 1.22 | 1.00 | 14 | TTCATATATAAAATAT | ATTGTTTTATATCTACAAT |
| | | NadR\_Enterobacteriales | 3 | 0.011 | 1.59 | 1.00 | 15 | TTCATATATAAAATAT | TGTTTAATATACTAAACA |
| | | NagC Ecol | 6 | 0.005 | 0.93 | 1.00 | 16 | TTCATATATAAAATAT | TATTTTTCGATAGATAAAAAAAG |
| | | torR | 3 | 0.005 | 0.32 | 0.64 | 7 | TTCATATATAAAATAT | CTGTTCATAT |
| 41 | | arcA | 3 | 0.001 | 0.08 | 0.15 | 12 | AATTATATTATAAA | GTTAATTAAATGTTA |
| | | PhoP Bsub | 12 | 0.020 | 3.79 | 1.00 | 14 | AATTATATTATAAA | TGATTAAAAAAGAAATTTAAAATATAATACA |
| | | rpoD16 | -1 | 0.005 | 0.34 | 0.33 | 13 | AATTATATTATAAA | ATTATACAGAAAAAATAAAAAATGTCAA |
| | | rpoD17 | -1 | 0.011 | 0.71 | 0.38 | 13 | AATTATATTATAAA | ATTATAACAAAAACTAAAGAATTTGTCAA |
| | | rpoD19 | -1 | 0.011 | 0.76 | 0.38 | 13 | AATTATATTATAAA | ATTATACCTCAAAACAGATAAAAAATGTCAA |
| | | XylR\_Gammaproteobacteria | 12 | 0.025 | 3.48 | 1.00 | 14 | AATTATATTATAAA | ACGAAAAATCGTAATAGAAGTGTAAAATAACATAATTG |
| 46 | | Hpr Bsub | 0 | 0.001 | 0.12 | 0.25 | 16 | ATAATATAATAATAAA | AAAATAGCATAATATAAAA |
| | | ihf | 5 | 0.010 | 0.69 | 0.69 | 16 | ATAATATAATAATAAA | CTAAGATAATAAAATGTTAAAAAATCAATAAGTTATATTTAATAAGTA |
| | | lrp | 7 | 0.001 | 0.08 | 0.16 | 16 | ATAATATAATAATAAA | AAAATGAATAATAAAAAAATCAAAT |
| | | MngR Ecol | -1 | 0.002 | 0.38 | 0.38 | 14 | ATAATATAATAATAAA | TAATACAAATACAA |
| | | XylR\_Gammaproteobacteria | 10 | 0.004 | 0.55 | 1.00 | 16 | ATAATATAATAATAAA | ACGAAAAATCGTAATAGAAGTGTAAAATAACATAATTG |
| 51 | | arcA | 1 | 0.003 | 0.20 | 0.40 | 14 | ATAATAATATTATAA | GTTAATTAAATGTTA |
| | | ArcA\_Gammaproteobacteria | -1 | 0.012 | 1.62 | 1.00 | 14 | ATAATAATATTATAA | TAACATTTAGTTAAC |
| | | DeoR\_Enterobacteriales | -1 | 0.021 | 2.96 | 1.00 | 14 | ATAATAATATTATAA | TGTTAGTATTCTAACA |
| | | Fur Hpyl | 1 | 0.008 | 1.51 | 0.49 | 15 | ATAATAATATTATAA | TATTATTTTGTTTTAATGAAAAT |
| | | MngR Ecol | -1 | 0.004 | 0.81 | 0.49 | 14 | ATAATAATATTATAA | TAATACAAATACAA |
| | | RcsA-RcsB Ecol | -1 | 0.008 | 1.45 | 0.49 | 14 | ATAATAATATTATAA | TAAGAATATTCCTA |
| | | XylR\_Gammaproteobacteria | 10 | 0.014 | 2.00 | 1.00 | 15 | ATAATAATATTATAA | ACGAAAAATCGTAATAGAAGTGTAAAATAACATAATTG |
| | | YhiX Ecol | 5 | 0.005 | 0.90 | 0.49 | 13 | ATAATAATATTATAA | TTAGGATATTTTTTTTAT |
| 52 | | arcA | -1 | 0.009 | 0.61 | 0.68 | 15 | ATAAAATAAAATTAAA | TAACATTTAATTAAC |
| | | ArcA\_Gammaproteobacteria | -1 | 0.013 | 1.90 | 1.00 | 15 | ATAAAATAAAATTAAA | TAACATTTAGTTAAC |
| | | CovR Spyo | -6 | 0.007 | 1.26 | 1.00 | 10 | ATAAAATAAAATTAAA | TTAAATTAAAA |
| | | lrp | 0 | 0.010 | 0.68 | 0.68 | 16 | ATAAAATAAAATTAAA | AAAATGAATAATAAAAAAATCAAAT |
| | | MtaR\_Streptococcaceae | 1 | 0.014 | 2.00 | 1.00 | 16 | ATAAAATAAAATTAAA | TATAGTTTAAAACTATA |
| 53 | | CodY Bsub | 33 | 0.004 | 0.78 | 0.78 | 14 | AATTATAATAAAAT | ATCTGTTCATGTAAAACGAAAGGTAGAAAGAAAAAAAAAAATAAAAACAACGAATAAAGAGACAA |
| | | CovR Spyo | -2 | 0.002 | 0.36 | 0.72 | 11 | AATTATAATAAAAT | TTAAATTAAAA |
| | | lrp | 3 | 0.022 | 1.51 | 1.00 | 14 | AATTATAATAAAAT | AAAATGAATAATAAAAAAATCAAAT |
Supplementary Table 5
