# Supplementary material for: Clostridium thermocellum transcriptomic profiles after exposure to furfural or heat stress
Source: Biotechnol Biofuels. 2013 Sep 12;6:131. doi: 10.1186/1754-6834-6-131 (PMC3848806; doi:10.1186/1754-6834-6-131)
Supplement: Additional file 4: Table S6. — Primer sequences used for Real Time qPCR validation of the microarray results. [file 1754-6834-6-131-S4.docx]

| **Primer name** | **Gene Target** | **Primer Sequence** |
| --- | --- | --- |
| Cthe_0197_F | Cthe_0197 | TGACGGTGATGTGGCTAAAG |
| Cthe_0197_R | Cthe_0197 | TCCCTTGGTCTTTTTCGTTG |
| Cthe_0482_F | Cthe_0482 | GAGCAGGGATTGGTAATGGA |
| Cthe_0482_R | Cthe_0482 | TACCGCAAGACCTACAAGCA |
| Cthe_0665_F | Cthe_0665 | GGAATTCAGGTGCTGGATGT |
| Cthe_0665_R | Cthe_0665 | GTCTCCTTGCTCTGCTTTGC |
| Cthe_1604_F | Cthe_1604 | GTGTCCCCGCTATTGCTAAA |
| Cthe_1604_R | Cthe_1604 | ATGGGTAAAATGCCGAATGA |
| Cthe_2531_F | Cthe_2531 | CGGAAAGGACATTGTCATCC |
| Cthe_2531_F | Cthe_2531 | CAAAGCCAGGGTTACGACAT |
| Cthe_2784_F | Cthe_2784 | CTGGTAAAAATAATGCTGGA |
| Cthe_2784_R | Cthe_2784 | TAATTTTTGCATTGACTTCC |
| Cthe_2801_F | Cthe_2801 | GTTTGGTTTGCCCGTAAGAA |
| Cthe_2801_R | Cthe_2801 | ACCAAAAGAGGGGAATGACC |

Supplementary Table 6: Primer sequences used in for Real Time qPCR validation of the microarray results.
